# NAT10-mediated lipid metabolic reprogramming drives EGFR-TKI resistance in non-small cell lung cancer via ac4C-dependent mRNA stabilization

**DOI:** 10.1186/s40164-025-00721-9

**Published:** 2025-11-27

**Authors:** Shuai Fang, Yuchao Zhu, Wei Chen, Wei Mao, Yuan Fang, Ziyuan Chen, Zhiqi Hong, Xiaodong Zhao, Wenmin Su, Yuning Pan, Guangyu Yao, Jianhua Wang, Chengwei Zhou

**Affiliations:** 1 Department of Thoracic Surgery, The First Affiliated Hospital of Ningbo University, Ningbo, Zhejiang Province People’s Republic of China; 2 Department of Radiology, The First Affiliated Hospital of Ningbo University, Ningbo, Zhejiang Province People’s Republic of China; 3 Ningbo University Health Science Center, Ningbo, Zhejiang Province People’s Republic of China; 4 Department of Radiology, The First Affiliated Hospital of Xiamen University, Xiamen, Fujian Province People’s Republic of China; 5 Department of Liver Surgery, Key Laboratory of Carcinogenesis and Cancer Invasion (Ministry of Education), Zhongshan Hospital, Liver Cancer Institute, Fudan University, Shanghai, People’s Republic of China; 6 Department of Thoracic Surgery, Zhongshan Hospital, Fudan University, Shanghai, People’s Republic of China; 7 Department of Shanghai Lung Cancer Center, Shanghai Chest Hospital, School of Medicine, Shanghai Jiao Tong University, Shanghai, People’s Republic of China

**Keywords:** NSCLC, EGFR-TKI resistance, NAT10, Lipid metabolism reprogramming, ac4C modification

## Abstract

The development of resistance to epidermal growth factor receptor (EGFR) tyrosine kinase inhibitor (TKI) treatment represents a significant challenge to targeted therapies for lung cancer. To explore the feasibility of epigenetic therapy in overcoming resistance, an epigenetic drug library was screened, identifying Remodelin as a potent enhancer of EGFR-TKI sensitivity in non-small cell lung cancer (NSCLC) cells. We demonstrated that the cytidine acetyltransferase NAT10 was overexpressed in NSCLC tissues and was associated with poor patient prognosis. NAT10 knockdown inhibited proliferation, increased apoptosis, and enhanced sensitivity to EGFR-TKIs both in vitro and in vivo. Mechanistically, NAT10 promoted EGFR-TKI resistance in NSCLC by remodeling fatty acid metabolism. Specifically, NAT10 was found to promote ac4C modification of fatty acid transport protein 4 (FATP4) and carnitine palmitoyltransferase 1 A (CPT1A) mRNAs, leading to increased stability and expression of these genes. Furthermore, p300-mediated H3K27ac acetylation was found to be a critical upstream regulator of NAT10 transcription. In vivo, mouse xenograft models confirmed that Remodelin significantly enhanced the antitumor efficacy of gefitinib. These findings suggest the potential of NAT10 as a therapeutic target to overcome EGFR-TKI resistance and improve treatment outcomes in patients with NSCLC.

**Supplementary Information:**

The online version contains supplementary material available at 10.1186/s40164-025-00721-9.

## Introduction

Non-small cell lung cancer (NSCLC) is a highly significant contributor to cancer-related mortality worldwide, accounting for a substantial proportion of all cancer deaths [1, 2]. Although targeted treatments like epidermal growth factor receptor tyrosine kinase inhibitors (EGFR-TKIs) have demonstrated promise in treating NSCLC cases with particular EGFR mutations, their long-term efficacy is severely limited by the emergence of acquired resistance, which is frequently caused by the T790M mutation in EGFR [3–5]. This acquired resistance poses a major therapeutic challenge in NSCLC treatment, ultimately leading to rapid disease progression, increased tumor aggressiveness, and significantly poorer patient outcomes [6–8]. The processes contributing to EGFR-TKI resistance and the development of therapies are still poorly understood despite advancements in our understanding of the genetic pathways underpinning resistance [9, 10]. As a result, the establishment of long-lasting and highly effective treatment plans necessitates a comprehensive understanding of the molecular mechanisms underlying EGFR-TKI resistance.

A conserved RNA modification observed in many RNA species, N4-acetylcytidine (ac4C), is essential for controlling RNA stability, translation, and RNA-protein interactions [11–13]. The acetyltransferase NAT10, which plays a significant role in cancer biology, catalyzes this specific molecular alteration [14, 15]. In malignancies such as hepatocellular carcinoma, colorectal cancer, and breast cancer, abnormal expression of NAT10 has been associated with tumor progression by promoting cell proliferation, migration, and invasion [16, 17]. Despite the growing understanding of the role of NAT10 in cancer, its specific effects on developing resistance to targeted therapies, such as EGFR-TKIs in NSCLC, remain unexplored. RNA modifications, including ac4C, have been implicated in the modulation of gene expression and cellular responses to therapy, suggesting that dysregulated NAT10-mediated ac4C modifications could contribute to EGFR-TKI resistance. Further research is required to elucidate this potential link, as uncovering how NAT10 influences drug resistance mechanisms could offer new strategies for overcoming therapeutic challenges in NSCLC.

One well-known characteristic of cancer is metabolic reprogramming, in which tumor cells modify their metabolic processes to continue growing, resist stress, and fight against reprogramming by treatment [18–20]. These modifications, which are crucial for tumor growth and increase resistance to targeted treatments like EGFR-TKIs, include changes in glycolysis, oxidative phosphorylation, and lipid metabolism in NSCLC. Recent research has suggested that epigenetic modifications, including RNA modifications such as ac4C, significantly drive these metabolic shifts [21, 22]. NAT10, responsible for catalyzing ac4C, has been implicated in multiple cancers and is thought to promote therapeutic resistance by facilitating metabolic reprogramming. It remains uncertain whether NAT10 modulates drug resistance via its direct or indirect impact on various metabolic pathways, necessitating comprehensive future research to elucidate the underlying molecular mechanisms.

The present study indicated that NAT10-catalyzed ac4C alteration is essential for conferring resistance to EGFR-TKI treatment in NSCLC. NAT10 promoted resistance through ac4C-mediated reprogramming of lipid metabolism, and combining NAT10 inhibition with EGFR-TKIs significantly improved therapeutic outcomes. The role of p300-mediated H3K27ac in regulating NAT10 transcription was also elucidated, highlighting the upstream mechanisms driving NAT10 overexpression in NSCLC. The findings revealed a potential mechanism of TKI resistance via the NAT10-ac4C-FATP4-CPT1A axis and indicated that targeting NAT10 with Remodelin may effectively address clinical EGFR-TKI resistance in NSCLC.

## Results

### Screening and verification of remodelin, identified from an epigenetic drug library, in enhancing the sensitivity of NSCLC to EGFR-TKI therapy

**Fig. 1:**
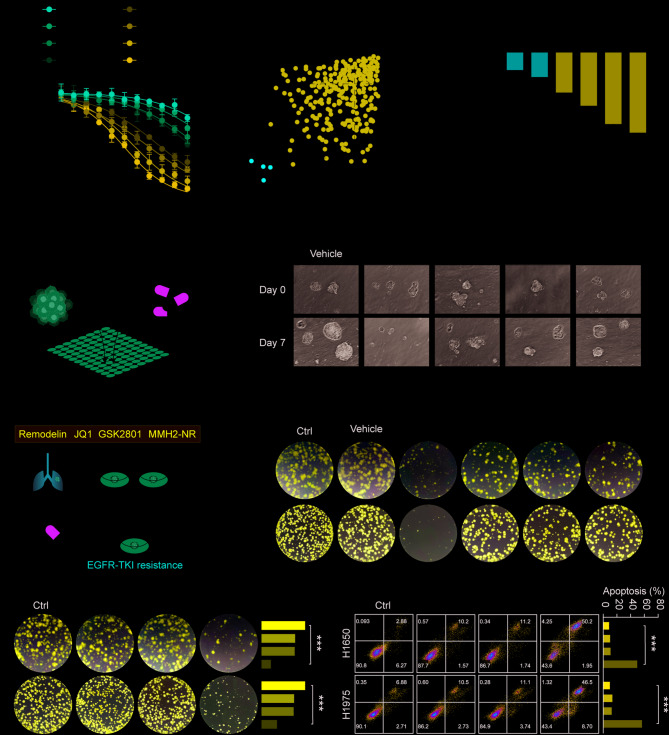
Remodelin is a sensitizer for NSCLC-TKI therapy. (A) The impact of various gefitinib concentrations (0–10 µM) on the viability of NSCLC cells was determined using CCK-8 assays. (B) The heatmap shows the synergistic effects of 168 epigenetic factor inhibitors (at their respective IC20 concentrations) combined with gefitinib (5 µM) or osimertinib (5 µM) on the viability of NSCLC cells, as measured by CCK-8 assays after 48 h of treatment. (C) The effect of 5 µM gefitinib on the growth of NSCLC organoids after 7 days of treatment. (D) The flowchart illustrates the screening process for TKI sensitizers, starting with an initial screening of 168 epigenetic factor inhibitors and culminating in identifying Remodelin as a promising candidate. (E-F) The effect of 5 µM gefitinib combined with IC20 concentrations of the indicated inhibitors on the growth of NSCLC-1-1 organoids and NSCLC cells after 7 days of treatment. (G-H) Clonogenic assays and flow cytometry analysis were used to assess the effects of treatment with gefitinib (5 µM) and Remodelin (10 µM) and their combination on the proliferation and apoptosis of resistant cells. Data are presented as means ± SD and are representative of three independent experiments, two-tailed unpaired Student’s t-test.***P < 0.001; **P < 0.01; *P < 0.05

Dose-response screening of NSCLC cell lines was used to find cells that were naturally resistant to gefitinib and osimertinib, evaluating both short-term viability and long-term clonogenic activity to explore the mechanisms underlying resistance to EGFR-TKI therapy in NSCLC (Fig. 1A and S1A-C). Gefitinib and a library of 168 epigenetic factor inhibitors were then administered to the resistant H1975 and H1650 cell lines to find substances that would increase their susceptibility to EGFR-TKIs. This led to the identification of four epigenetic inhibitors that significantly increased the sensitivity of both H1975 and H1650 cells to gefitinib (Fig. 1B). To verify these findings, NSCLC organoids were developed and treated with gefitinib to identify the most drug-resistant organoid models (Fig. 1D). After the selection of resistant NSCLC organoids, these were treated, in parallel with different NSCLC cell lines, with 5 µM gefitinib or osimertinib in combination with each of the epigenetic inhibitors at its IC20 concentration (Fig. 1C and S1D). The findings demonstrated that NSCLC-1-1 organoids and NSCLC cells were significantly more sensitive to gefitinib and osimertinib when the epigenetic inhibitor Remodelin was present (Fig. 1E-F and S1E-F). This combination therapy significantly decreased the number of NSCLC cells that formed colonies and halted the growth of the NSCLC-1-1 organoids.

In contrast, the control group and the vehicle-only group showed no significant variations, indicating that the vehicle itself did not influence the outcome, and Remodelin alone did not affect cell growth. Combining gefitinib or osimertinib with Remodelin inhibited colony formation in HCC2279 and PC-9/GR cells (Fig. 1G and S1G). Treatment with Remodelin and either osimertinib or gefitinib resulted in a marked elevation of apoptosis rates in H1650 and H1975 cells, as shown by flow cytometry analysis (Fig. 1H and S1H). These findings suggest that Remodelin substantially enhances the responsiveness of both NSCLC organoids and cell lines to treatment with gefitinib or osimertinib.

### Upregulation of NAT10 is strongly linked to malignant characteristics and a poor prognosis in NSCLC

**Fig. 2:**
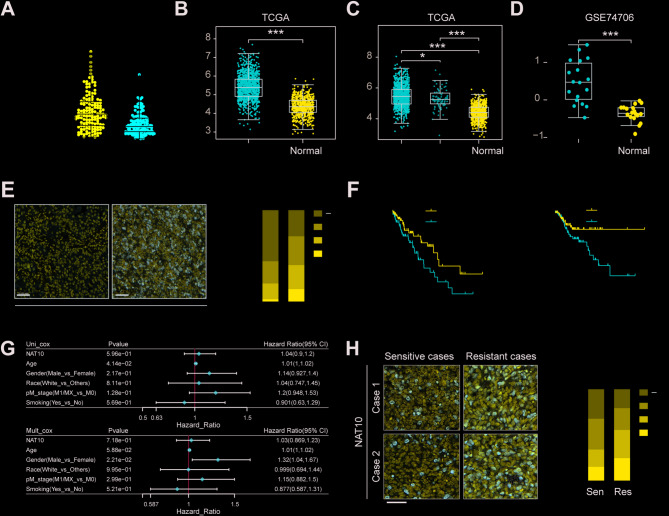
Expression levels of NAT10 in NSCLC tissues and its clinical significance. (A) NAT10 mRNA expression levels in 138 paired NSCLC tumors and adjacent normal tissue samples measured by qRT-PCR. (B-D) Comparison of NAT10 mRNA expression levels between NSCLC tumor and adjacent normal lung tissues using data from TCGA and GEO. (E) Immunohistochemical analysis of NAT10 protein expression in NSCLC tumor tissues and adjacent normal tissues. (F) Kaplan-Meier survival analysis of the association between NAT10 expression and overall survival and disease-free survival in patients with NSCLC. (G) Multivariate Cox regression analysis of overall and disease-free survival concerning NAT10 expression and other clinicopathological variables. (H) Representative images of immunohistochemical staining of NAT10 in tissues from NSCLC patients with acquired resistance or sensitivity to gefitinib or osimertinib. Data are presented as means ± SD and are representative of three independent experiments, two-tailed unpaired Student’s t-test. ***P < 0.001; **P < 0.01; *P < 0.05

It has been previously reported that Remodelin, an inhibitor of NAT10, can enhance cancer cell susceptibility to therapeutic agents [9, 23]. Drug-resistant and drug-sensitive cell lines and patient tissues were used to test the expression of NAT10 to learn more about its function in NSCLC drug resistance. There may be a connection between NAT10 expression and the emergence of drug resistance, as evidenced by the significantly higher amounts of NAT10 mRNA and protein in drug-resistant NSCLC cell lines compared to their drug-sensitive counterparts (Figure S2B-C). Furthermore, NAT10 expression was significantly elevated in tumor tissues compared to neighboring non-cancerous tissues, according to qRT-PCR analysis of 138 pairs of NSCLC and adjacent normal tissue samples (Fig. 2A). Analysis of publically accessible information from the gene expression omnibus (GEO) database and the cancer genome atlas (TCGA) showed consistently elevated NAT10 expression in NSCLC tissues as well as in a variety of other cancer types, which is consistent with these findings (Fig. 2B-D and S2A). Immunohistochemical staining of NSCLC tissue sections further confirmed this observation, showing significantly higher NAT10 levels in tumor tissues than in surrounding non-tumor cells (Fig. 2E). Overall survival (OS) and disease-free survival (DFS) in patients with NSCLC were examined to evaluate the prognostic importance of NAT10. High NAT10 expression was shown to be substantially linked to worse DFS and OS by the Kaplan-Meier analysis (Fig. 2F).

Furthermore, it was confirmed by multivariate Cox regression analysis that poor clinical outcomes in NSCLC were independently predicted by higher NAT10 expression (Fig. 2G). In addition, compared to drug-sensitive patients, individuals with NSCLC who were resistant to gefitinib or osimertinib had considerably greater levels of NAT10 expression (Fig. 2H). Collectively, these findings indicate that NAT10 expression is significantly associated with the development of drug resistance and adverse clinical outcomes, such as advanced tumor stage and poor prognosis, in NSCLC.

### Knockdown of NAT10 inhibits cell proliferation and enhances sensitivity to TKI therapy

**Fig. 3:**
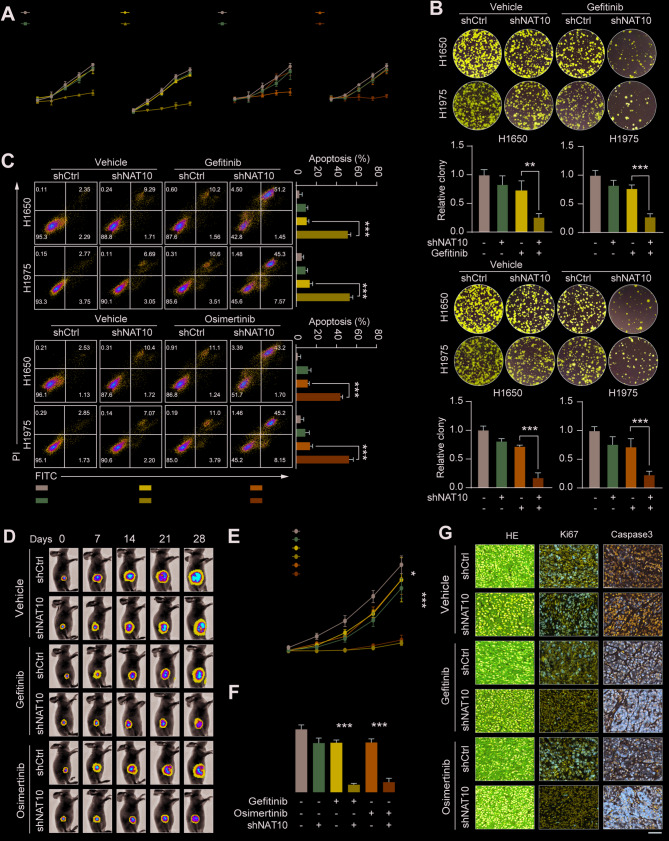
Knockdown of NAT10 inhibits cell proliferation and enhances sensitivity to TKI therapy. (A) Cell viability was assessed using CCK-8 assays after treatment periods of 0, 24, 48, 72, and 96 h with the specified treatments. (B) Colony formation was assessed as colony numbers after 12 days of culture. (C) Apoptosis in H1650 and H1975 cells after shRNA-mediated NAT10 knockdown and treatment with gefitinib or osimertinib, as analyzed by flow cytometry. (D) Representative images of xenograft tumors derived from shRNA-mediated NAT10-knockdown cells with treatment with gefitinib or osimertinib and control tumors without NAT10 knockdown. (E) Growth of xenograft tumors derived from shRNA-mediated NAT10-knockdown cells with treatment with gefitinib or osimertinib and control tumors without NAT10 knockdown. (F)Weights of xenograft tumors derived from shRNA-mediated NAT10-knockdown cells with treatment with gefitinib or osimertinib and control tumors without NAT10 knockdown. (G) Immunohistochemical images of xenograft tumors derived from shRNA-mediated NAT10 knockdown cells treated with gefitinib or osimertinib, as well as the immunohistochemical images of control tumors without NAT10 knockdown. Data are presented as means ± SD and are representative of three independent experiments, two-tailed unpaired Student’s t-test.***P < 0.001; **P < 0.01; *P < 0.05

To investigate the impact of NAT10 on EGFR-TKI resistance in NSCLC cells, shRNA targeting NAT10 was established in the two most resistant cell lines, H1650 and H1975, to reduce its expression and determine whether decreased NAT10 expression would attenuate EGFR-TKI resistance. Following shRNA treatment, NAT10 expression was significantly decreased, according to Western blot (WB) and qRT-PCR studies (Figure S3A-B). The NAT10 deletion dramatically reduced the IC50 values of gefitinib or osimertinib in H1659 and 1975 cells, according to CCK-8 analysis (Figure S3C). Cell viability was evaluated using CCK-8 assays, and the long-term proliferative potential was examined using colony formation assays. The results showed that cell viability and long-term proliferative capacity decreased after the combined treatment (Fig. 3A-B).

Furthermore, flow cytometry and WB were used to assess the apoptotic rates in NAT10-knockdown H1975 and H1650 cells treated with either osimertinib or gefitinib (Fig. 3C and S3D). The findings demonstrated that NAT10 knockdown significantly raised NSCLC cells’ susceptibility to gefitinib and osimertinib. A subcutaneous xenograft model was created by injecting mice with either control or shNAT10-treated NSCLC cells to evaluate the effects of NAT10 knockdown in vivo. After NAT10 knockdown, the mice received oral gefitinib or osimertinib treatment, and tumor growth was routinely observed. The results demonstrated that gefitinib and osimertinib greatly inhibited tumor growth (Fig. 3D-F). The tumor tissues were subsequently investigated using H&E staining and Ki-67 immunohistochemical analysis, which showed that NAT10 knockdown significantly increased the therapeutic efficacy of gefitinib and Osimertinib (Fig. 3G). These findings suggest that EGFR-TKI resistance is largely caused by NAT10 and that EGFR-TKI treatments may be more effective in NSCLC if NAT10 is targeted.

### NAT10 regulates lipid metabolic reprogramming in NSCLC

**Fig. 4:**
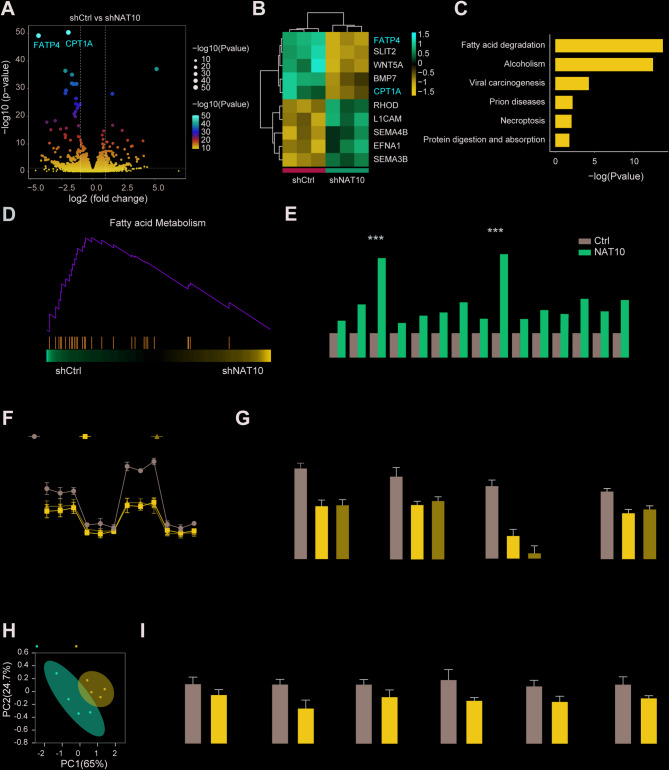
NAT10 regulates lipid metabolism reprogramming in NSCLC. (A) Volcano plot showing differentially expressed genes between NAT10-knockdown and control cells in H1650 cells. (B) Heatmap illustrating the expression of differentially expressed genes involved in lipid metabolism. (C-D) Pathway enrichment of differentially expressed genes, shown by GSEA and KEGG analyses. (E) mRNA expression of lipid metabolism-associated genes following NAT10 overexpression in H1650 cells, as measured by qRT-PCR. (F-G) OCR, basal respiration, maximal respiration, ATP production, and spare respiratory capacity values. (H-I) Changes in metabolite levels following NAT10 knockdown, as shown by metabolomics analysis. Data are presented as means ± SD and are representative of three independent experiments, two-tailed unpaired Student’s t-test. ***P < 0.001; **P < 0.01; *P < 0.05

To further clarify the role of NAT10 in the pathogenesis and progression of NSCLC, the transcriptomes of NSCLC cells with NAT10 knockdown were sequenced. This revealed that NAT10 knockdown resulted in the downregulation of multiple lipid metabolism-related genes, building on the finding that NAT10 knockdown increases the sensitivity of NSCLC cells to EGFR-TKI treatment by inhibiting cell proliferation and promoting apoptosis (Fig. 4A-B). GSEA and KEGG enrichment analyses suggest that abnormal expression levels of NAT10 were associated with the fatty acid metabolism pathway, implying that NAT10 may regulate lipid metabolism in NSCLC cells (Fig. 4C-D). QRT-PCR analysis of genes involved in lipid metabolic pathways was conducted in H1650 cells after NAT10 overexpression to confirm the observed alterations in lipid metabolism. The expression of these genes was found to be significantly increased, supporting the hypothesis that NAT10 regulates lipid metabolism (Fig. 4E).

NAT10 knockdown significantly lowered the oxygen consumption rate (OCR), basal respiration, ATP generation, maximal respiration, and spare respiratory capacity in both H1650 and H1975 cells, according to additional studies using a cell flux analyzer. This suggests impaired mitochondrial function and overall cellular metabolism (Fig. 4F-G and S4C-D). The results indicated that NAT10 is essential for properly regulating lipid metabolism and energy homeostasis in NSCLC cells. Further evidence for the function of NAT10 in controlling metabolic processes came from a metabolomics study of H1650 cells, which showed changed amounts of several metabolic products (Fig. Fig. 4H-I). Furthermore, lipid droplet staining of H1650 and H1975 cells indicated reduced lipid uptake following NAT10 knockdown (Figure S4A), and measurements of free fatty acids showed increased levels, confirming the disruption of lipid metabolism (Figure S4B). The above findings imply that NAT10 is essential for preserving energy generation and lipid metabolism in NSCLC cells and that its disruption may increase EGFR-TKI sensitivity.

### NAT10 regulates the stability of FATP4 and CPT1A mRNAs through ac4C modification

**Fig. 5:**
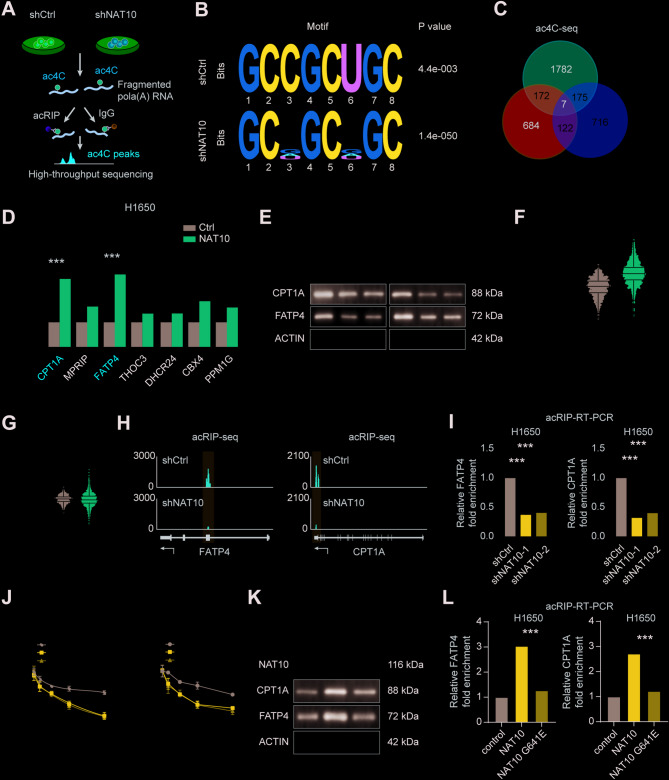
NAT10 regulates the stability of FATP4 and CPT1A mRNAs via ac4C modification. (A) Schematic representation of the acRIP-seq workflow in H1975 cells. (B) Consensus motif analysis of ac4C-containing RNA sequences in control and NAT10-knockdown H1975 cells. (C) Venn diagram shows the overlap between differentially expressed genes identified by acRIP-seq following NAT10 knockout and those identified by RNA-seq following NAT10 knockout or overexpression. (D) qRT-PCR verification of the expression of differentially expressed genes common to the acRIP-seq and RNA-seq datasets after NAT10 overexpression. (E) Western blot analysis showing the protein expression levels of FATP4 and CPT1A after NAT10 knockdown. (F-G) Expression levels of FATP4 and CPT1A in NSCLC tissues compared to normal lung tissues, based on data from the TCGA dataset. (H) IGV tracks displaying NAT10 binding regions and ac4C peaks on FATP4 and CPT1A mRNAs. (I) acRIP-qPCR analysis showing the enrichment of FATP4 and CPT1A transcripts in NAT10-knockdown cells. (J) Time-course analysis of FATP4 and CPT1A mRNA expression levels following treatment with actinomycin D to assess mRNA stability. (K) Western blotting analysis of FATP4 and CPT1A protein expression in cells overexpressing wild-type NAT10 or the catalytically inactive NAT10 G641E mutant. (L) an acRIP-qPCR analysis demonstrating the enrichment of FATP4 and CPT1A mRNAs in cells overexpressing wild-type NAT10 or the NAT10 G641E mutant. Data are presented as means ± SD and are representative of three independent experiments, two-tailed unpaired Student’s t-test. ** and CPT1A mRNAs via ac4C modification. (A) Schematic representation of the acRIP-seq workflow in H1975 cells. (B) Consensus motif analysis of ac4C-containing RNA sequences in control and NAT10-knockdown H1975 cells. (C) Venn diagram shows the overlap between differentially expressed genes identified by acRIP-seq following NAT10 knockout and those identified by RNA-seq following NAT10 knockout or overexpression. (D) qRT-PCR verification of the expression of differentially expressed genes common to the acRIP-seq and RNA-seq datasets after NAT10 overexpression. (E) Western blot analysis showing the protein expression levels of FATP4 and CPT1A after NAT10 knockdown. (F-G) Expression levels of FATP4 and CPT1A in NSCLC tissues compared to normal lung tissues, based on data from the TCGA dataset. (H) IGV tracks displaying NAT10 binding regions and ac4C peaks on FATP4 and CPT1A mRNAs. (I) acRIP-qPCR analysis showing the enrichment of FATP4 and CPT1A transcripts in NAT10-knockdown cells. (J) Time-course analysis of FATP4 and CPT1A mRNA expression levels following treatment with actinomycin D to assess mRNA stability. (K) Western blotting analysis of FATP4 and CPT1A protein expression in cells overexpressing wild-type NAT10 or the catalytically inactive NAT10 G641E mutant. (L) an acRIP-qPCR analysis demonstrating the enrichment of FATP4 and CPT1A mRNAs in cells overexpressing wild-type NAT10 or the NAT10 G641E mutant. Data are presented as means ± SD and are representative of three independent experiments, two-tailed unpaired Student’s t-test. ***P < 0.001; **P < 0.01; *P < 0.05

Building upon previous findings linking NAT10 and ac4C modification to drug resistance in NSCLC, the molecular mechanisms underlying these observations were investigated. It was hypothesized that NAT10 regulates lipid metabolism by modulating the stability of key metabolism-associated mRNAs through ac4C modification. NAT10-knockdown NSCLC cells were analyzed by acRIP-seq (Fig. 5A-C, S5A) to investigate this. After overexpressing NAT10, qRT-PCR analysis revealed that genes related to fatty acid oxidation (FAO) and lipid uptake were significantly upregulated (Fig. 5D and S5B). WB confirmed this at the protein level as well, demonstrating that NAT10 knockdown reduced the expression of carnitine palmitoyltransferase 1 A (CPT1A) and FATP4 (Fig. 5E). Furthermore, qRT-PCR research revealed that tumor tissues had significantly higher FATP4 and CPT1A expression levels than neighboring non-cancerous tissues (Figure S5C). The data from the TCGA database also confirmed this result (Fig. 5F-G). Pearson correlation analysis indicated a positive correlation between NAT10 expression and the expression levels of FATP4 and CPT1A in NSCLC tissues. (Figure S5D-E). To determine whether NAT10 regulates mRNA stability through ac4C modification, we performed an integrative genomics viewer (IGV) analysis of the acRIP-seq data. After the knockdown of NAT10, the ac4C enrichment in the CDS region of FATP4 mRNA and the 3′ UTR region of CPT1A mRNA was significantly decreased. (Fig. 5H), these ac4C sites may play a dynamic role in regulating mRNA stability. The acRIP-qRT-PCR results further supported this observation, demonstrating that NAT10 knockdown led to the downregulation of FATP4 and CPT1A expression (Fig. 5I). Actinomycin D, a transcriptional inhibitor that blocks new mRNA synthesis, was then used to assess the mRNA stability, showing significant reductions in the stability of FATP4 and CPT1A mRNAs over time following NAT10 knockdown (Fig. 5J and S5F), suggesting that NAT10 may enhance the stability of these transcripts through ac4C modification. To confirm the role of ac4C modification in mediating mRNA stability, firefly luciferase reporter genes containing either the wild-type or a mutant of FATP4 and CPT1A were constructed. The previously identified ac4C sites in the mutant were altered by substituting cytidine (C) with thymidine (T). The dual-luciferase assay showed that overexpression of NAT10 significantly increased luciferase activity in the wild-type, whereas there was no significant change in luciferase activity upon NAT10 overexpression in the mutant type. This indicates a direct interaction between NAT10 and FATP4 (Figure S5G). In addition, overexpressing a catalytically inactive NAT10 G641E mutant did not stabilize FATP4 and CPT1A mRNAs or enhance ac4C enrichment (Fig. 5K-L and S5H), demonstrating the crucial role that ac4C modification plays in the stability of these transcripts. When considered collectively, our findings clearly show that NAT10 increases the translation and expression of the encoded proteins by improving the stability of FATP4 and CPT1A mRNA through ac4C modification, thereby enhancing lipid metabolism.

### NAT10 promotes TKI resistance in NSCLC cells by regulating FATP4 and CPT1A

**Fig. 6:**
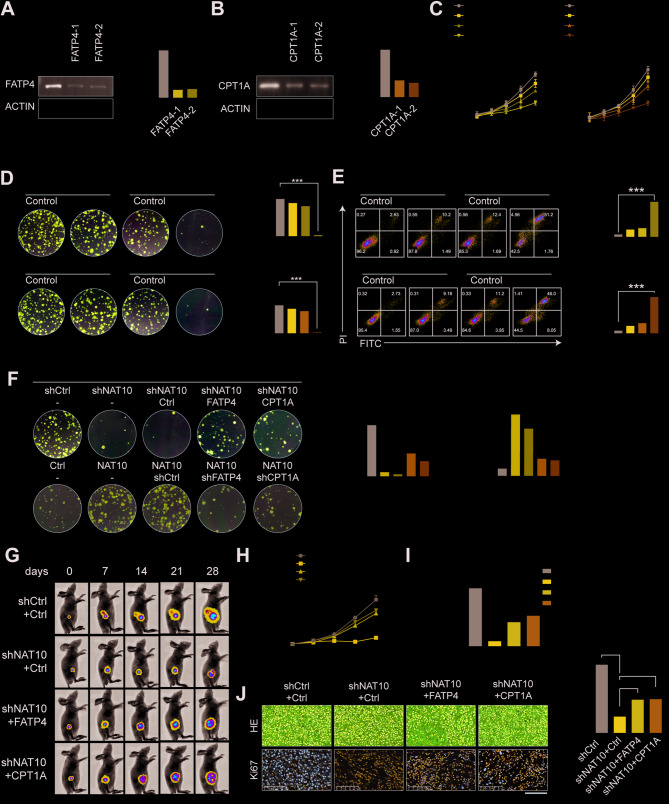
NAT10 promotes TKI resistance in NSCLC cells by regulating FATP4 and CPT1A. (A-B) Verification of FATP4 knockdown efficiency in H1650 cells transfected with shRNA targeting FATP4 or CPT1A, using qRT-PCR and Western blotting. (C-D) The effects of FATP4 or CPT1A knockdown on the proliferation of H1650 cells treated with gefitinib were assessed using cell viability and colony formation assays, respectively. (E) Flow cytometry analysis results of apoptosis rates in H1650 cells with FATP4 knockdown or CPT1A knockdown, with or without treatment with gefitinib. (F) Colony formation assay results evaluating the proliferative capacity of H1650 cells with simultaneous NAT10 knockdown and overexpression of FATP4 or CPT1A, as well as PC9 cells with overexpression of NAT10 and knockdown of FATP4 or CPT1A. (G-I) Assessment of tumor growth in a subcutaneous xenograft model using H1975 cells with different genetic manipulations. (J) Immunohistochemical examination of xenograft tumor sections and representative pictures of H&E-stained sections display the tumor’s shape and the expression of pertinent markers. Data are presented as means ± SD and are representative of three independent experiments, two-tailed unpaired Student’s t-test. ***P < 0.001; **P < 0.01; *P < 0.05

We investigated how NAT10 promoted TKI resistance in NSCLC cells by regulating the key metabolic enzymes FATP4 and CPT1A. FATP4 and CPT1A were successfully knocked down in H1650 cells using specific shRNAs (shFATP4 and shCPT1A, respectively), and qRT-PCR and WB analyses confirmed reductions in their expression (Fig. 6A-B). Knockdown of FATP4 or CPT1A markedly increased the susceptibility of NSCLC cells to osimertinib and gefitinib, according to subsequent CCK-8, colony formation experiments and flow cytometry (Fig. 6C-E). The regulatory role of NAT10 was further confirmed by rescue experiments, which demonstrated that the increase in TKI sensitivity was reversed in NAT10-knockdown cells by overexpressing either FATP4 or CPT1A. Conversely, overexpression of NAT10 in TKI-sensitive PC9 cells increased resistance to TKI drugs. Still, this effect was counteracted by the knockdown of either FATP4 or CPT1A, indicating that NAT10 promoted TKI resistance at least partly through these enzymes (Fig. 6F and S6A-C). In vivo, investigations employing a subcutaneous xenograft model demonstrated that the growth of tumors resulting from NAT10 knockdown cells paired with overexpression of FATP4 or CPT1A was considerably faster than tumors with NAT10 knockdown alone (Fig. 6G-I). However, treatment with the CPT1A inhibitor Etomoxir suppressed tumor growth, consistent with the in vitro findings (Figure S6D-F). H&E staining and immunohistochemical analysis further confirmed that FATP4 and CPT1A promoted tumor growth in vivo, consistent with the molecular biology results (Fig. 6J). The results show that NAT10 enhances TKI resistance in NSCLC cells by modulating FATP4 and CPT1A.

### p300-mediated H3K27ac activates NAT10 transcription in NSCLC

**Fig. 7:**
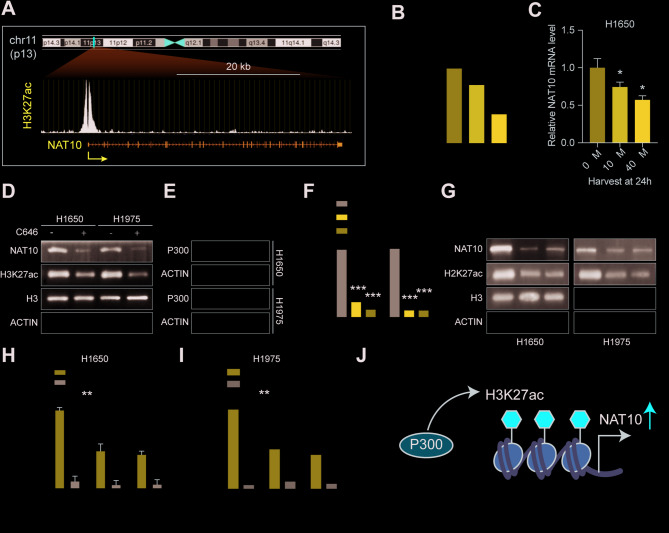
p300-mediated H3K27ac activates NAT10 transcription in NSCLC. (A) UCSC Genome Browser screenshot showing H3K27ac enrichment at the NAT10 promoter region in H1650 cells. (B) qRT-PCR analysis of NAT10 mRNA expression levels in H1650 cells treated with the p300 inhibitor C646 for the indicated times. (C) qRT-PCR analysis of NAT10 mRNA expression levels in H1650 cells treated with increasing concentrations of C646 for 24 h. (D) Western blots showing NAT10 and H3K27ac protein levels in H1650 and H1975 cells treated with C646 for 24 h. (E) Verification of p300 knockdown efficiency in H1650 and H1975 cells transfected with shRNA targeting p300, as assessed by Western blots showing p300 protein expression. (F) qRT-PCR analysis of NAT10 mRNA expression levels in H1650 cells after p300 knockdown. (G) Western blots show NAT10 and H3K27ac protein levels in H1650 and H1975 cells after p300 knockdown. (H-I) ChIP assay results of p300 binding and H3K27ac enrichment at the NAT10 promoter region in H1650 and H1975 cells after p300 knockdown or control cells. (J) Schematic model illustrating the proposed mechanism by which p300-mediated H3K27ac modification activates NAT10 transcription. Data are presented as means ± SD and are representative of three independent experiments, two-tailed unpaired Student’s t-test. ***P < 0.001; **P < 0.01; *P < 0.05

The mechanisms driving NAT10 overexpression in NSCLC were analyzed further using the UCSC genome browser, revealing significant H3K27 acetylation (H3K27ac) enrichment within the NAT10 promoter and thus suggesting that chromatin acetylation may be a key regulator of NAT10 transcription (Fig. 7A). The p300/CBP complex is known to catalyze H3K27ac, so 138 pairs of NSCLC and normal tissue samples were investigated, finding that p300 mRNA expression was considerably elevated in NSCLC tissues (Figure S7A). This suggests that p300 represented a potential driver of NAT10 upregulation (Figure S7D). In order to investigate this hypothesis, C646, a particular inhibitor of p300’s histone acetyltransferase activity, was administered to NSCLC cells. This demonstrated significant reductions in NAT10 expression that were dose- and time-dependent (Fig. 7B-C and S7B-C). WB analysis confirmed that C646 treatment reduced H3K27ac and NAT10 protein levels (Fig. 7D). To directly assess the role of p300, p300 was knocked down using shRNA. WB showed that the p300 protein level was significantly reduced (Fig. 7E). Moreover, qRT-PCR analysis revealed that the NAT10 mRNA level significantly decreased after p300 knockdown (Fig. 7F). Furthermore, H3K27ac levels and NAT10 protein expression were also reduced after p300 knockdown (Fig. 7G), similar to the effects observed with the C646 treatment. ChIP assays further demonstrated enrichment of the NAT10 promoter region for p300 binding and H3K27ac signals. Notably, p300 knockdown significantly reduced the enrichment of H3K27ac within the NAT10 promoter (Fig. 7H-I), indicating that p300-mediated H3K27ac are crucial in activating NAT10 transcription. This suggests that p300-mediated H3K27ac is a key driver of NAT10 transcription in NSCLC (Fig. 7J).

### In vitro and in vivo, remodelin increases NSCLC’s susceptibility to gefitinib

**Fig. 8:**
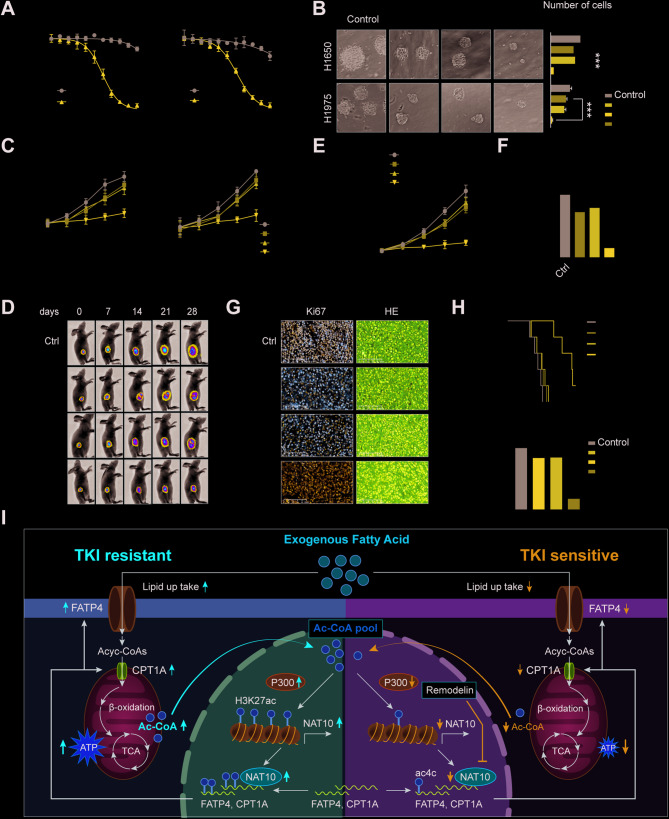
Remodelin enhances NSCLC sensitivity to gefitinib in vitro and *in vivo.* (A) CCK-8 assay results measuring cell viability following treatment with different concentrations of gefitinib in combination with Remodelin. (B) Soft agar colony formation assays evaluate cell proliferation after treatment with remodelin, gefitinib, or their combination. (C) CCK-8 assay results show proliferation of cells following treatment with gefitinib and/or Remodelin. (D-F) Tumor growth in subcutaneous xenograft model mice after treatment with Remodelin (100 mg/kg), gefitinib (25 mg/kg), or their combination. (G-H) H&E staining and immunohistochemical analysis of tumor tissues; survival outcomes in subcutaneous xenograft model mice following treatment with Remodelin, gefitinib, or their combination. (I) The role of NAT10 in NSCLC cancer. NAT10 enhances the stability of FATP4 and CPT1A mRNA through ac4C modification, promoting lipid metabolism and consequently increasing the resistance of NSCLC to EGFR-TKIs. Ac-CoA produced from lipid metabolism activates NAT10 transcription via P300-mediated H3K27ac. Data are presented as means ± SD and are representative of three independent experiments, two-tailed unpaired Student’s t-test. ***P < 0.001; **P < 0.01; *P < 0.05

The therapeutic potential of Remodelin, a NAT10 inhibitor used in conjunction with gefitinib, was then examined in light of the findings linking NAT10 to EGFR-TKI resistance. The efficacy of this combination was initially evaluated in NSCLC cells in vitro. Across various gefitinib concentrations, CCK-8 experiments showed that combining Remodelin and gefitinib significantly reduced cell viability compared to either drug alone (Fig. 8A). Further, the proliferative capacity of NSCLC cells treated with the combination was much lower than that of cells treated with a single drug, according to CCK-8, colony formation assays in soft agar, and EDU tests (Fig. 8B-C and S8A). Lipid droplet staining of H1650 and H1975 cells showed that the combination treatment significantly reduced lipid uptake in NSCLC cells compared to single-agent therapy (Figure S8B). A subcutaneous xenograft model was established in immunodeficient mice to confirm these findings in a preclinical model. Consistent with the in vitro results, the Remodelin combination with gefitinib led to significant tumor growth inhibition compared to gefitinib monotherapy (Fig. 8D-F). Histological analysis of tumor tissues using H&E staining and immunohistochemistry revealed reduced proliferation in the combination treatment group, further supporting the enhanced therapeutic effect of gefitinib after combination with Remodelin (Fig. 8G-H). Oxygen consumption rate (OCR) measurements were conducted to investigate the mechanism underlying this synergistic effect and assess mitochondrial function. The OCR, basal respiration, ATP production, maximal respiration, and spare respiratory capacity of H1650 and H1975 cells were significantly inhibited by the combination of Remodelin and gefitinib, indicating impaired mitochondrial function. The results were better than those obtained with gefitinib alone (Figure S8C-F). These results imply that remodelin increases the NSCLC cell’s sensitivity to gefitinib by interfering with mitochondrial and lipid metabolism (Fig. 8I). The results of this study suggest that by concurrently addressing lipid metabolism and mitochondrial function, the combination of remodelin and gefitinib may offer a viable treatment approach for overcoming TKI resistance in NSCLC.

## Discussion

One of the biggest challenges to the clinical management of NSCLC is resistance to EGFR-TKIs [24, 25]. While treatment with EGFR-TKIs can markedly extend patient survival, the evolution of resistance constrains their long-term effectiveness, underscoring the critical necessity to comprehend the underlying molecular pathways.

NAT10, the only known enzyme mediating RNA ac4C modifications, plays a key role in the onset and progression of various diseases, especially tumors [26–28]. NAT10 significantly improves tumor progression by controlling mRNA stability and translation and is linked to poor prognosis in multiple cancer types, including colorectal and bladder cancer [29, 30]. Moreover, the functions of NAT10 have been found to differ according to the cancer type. For instance, in liver cancer, NAT10 stimulates the acetylation of the ACLY protein’s K468 site, which raises acetyl-CoA synthesis and triggers the transcription of drug-resistant genes, leading to chemotherapy resistance [31]. NAT10 promotes cell proliferation in breast cancer by acetylating CEP170 mRNA to increase its translation efficiency [32]. According to the current study, NAT10 plays a crucial role in tumor resistance since its expression was much higher in EGFR-TKI-resistant NSCLC tissues and cells and increased resistance to EGFR-TKIs.

Another important finding was the identification of FATP4 and CPT1A as downstream targets of NAT10. FATP4 and CPT1A are integral to fatty acid metabolism’s absorption and oxidation mechanisms [33]. FATP4 is widely distributed in various tissues, where it is responsible not only for the uptake and transport of fatty acids but also for the function of acyl-CoA synthetase, which regulates tissue lipid homeostasis [34, 35]. CPT1A is an essential enzyme in fatty acid oxidation, facilitating the transport of long-chain fatty acids to the mitochondria for β-oxidation, producing ATP to energize the cell [33]. Research has shown that these enzymes are crucial for altering the lipid metabolism characteristic of cancer. By improving fatty acid uptake and oxidation, these processes promote the growth and survival of tumor cells while also influencing their immune microenvironment [36, 37]. A lack of FATP4 can lead to severe metabolic disorders and is closely related to abnormal lipid metabolism in tumors. At the same time, the upregulation of CPT1A is associated with the enhancement of energy metabolism in tumor cells and the development of drug resistance [38, 39]. The current study found that EGFR-TKI-resistant NSCLC cells have elevated expression levels of FATP4 and CPT1A. NSCLC cells’ sensitivity to EGFR-TKIs increased significantly when the expression of either enzyme was knocked down. Notably, NSCLC cells overexpressed NAT10, which developed EGFR-TKI resistance and was reversible by inhibiting FATP4 or CPT1A. These results indicate that lipid metabolism mediated by the NAT10/FATP4/CPT1A axis is one of the critical pathways affecting tumor cell resistance.

There is mounting evidence that the development of drug resistance is directly linked to metabolic reprogramming in tumor cells, particularly the augmentation of fatty acid oxidation [39, 40]. Fatty acid oxidation provides energy and metabolic intermediates for cells and is critically involved in epigenetic regulation through the generation of acetyl-CoA [41, 42]. Excessive acetyl-CoA can act as an acetyl donor to promote histone acetylation, especially H3K27ac acetylation mediated by p300, affecting chromatin structure and regulating gene expression [43, 44]. Several studies revealed that p300-mediated histone acetylation maintained the high expression level of NAT10 in NSCLC and that it was intimately linked to the control of lipid metabolism and EGFR-TKI resistance. The H3K27ac modification may activate NAT10-related signaling pathways, promote fatty acid oxidation, and stimulate tumor energy metabolism, ultimately exacerbating tumor cell resistance to EGFR-TKIs. This mechanism provides a new perspective for understanding drug resistance, offering additional critical insights and important molecular clues that will be useful for developing more effective future treatment strategies.

As a NAT10 inhibitor, remodelin has been shown to significantly affect cancer cells’ mitochondrial lipid metabolism through various intricate pathways, thereby altering the cells’ susceptibility to different chemotherapeutic drugs [38, 45]. Studies have shown that treatment of cancer cells with Remodelin markedly reduced the levels of total cholesterol and triglycerides, indicating that NAT10 plays an important role in maintaining mitochondrial fatty acid metabolism [32]. In addition to Remodelin, several other NAT10 inhibitors have demonstrated significant potential in cancer treatment, highlighting their therapeutic relevance and possible clinical applications [31, 46]. Further investigations have demonstrated that NAT10 activates the transcription of drug-resistant genes and promotes chemotherapy resistance by acetylating the K468 site of ACLY, enhancing acetyl-CoA synthesis [31]. According to the current study, NAT10 inhibitors and EGFR-TKIs might successfully reverse EGFR-TKI resistance, potentially improving the prognosis of patients with NSCLC.

## Conclusion

In conclusion, the results of this investigation identified NAT10 as a major regulator of lipid metabolism reprogramming and EGFR-TKI resistance in NSCLC. NAT10 has been shown to affect the stability and expression of genes involved in fatty acid metabolism via ac4C and H3K27ac changes. This revelation opens up new opportunities for developing anti-resistance treatment regimens and gives a new avenue for the future precision treatment of EGFR-TKI-resistant NSCLC.

## Materials and methods

### Cell culture and primary tissue samples

The cell lines H1650, H1975, HCC2279, HCC2935, HCC4006, and HCC827 were purchased from the ATCC (USA), and the PC-9 and PC-9/GR cell lines were obtained from the ECACC (London, UK). Every cell line was cultured in RPMI-1640 (Gibco, USA) with 10% fetal bovine serum (FBS, Gibco) and 1% penicillin-streptomycin (HYClone, USA) at 37 °C with 5% CO_2_ in a humidified incubator. A mycoplasma detection kit (Beyotime, China) was used to check the cells for mycoplasma contamination regularly, and all cultures turned out negative. Ningbo University’s First Affiliated Hospital provided the NSCLC tissue samples for this investigation. The First Affiliated Hospital of Ningbo University’s Institutional Review Board approved this study (Approval No. 2023–220 A-02), which complied with the Declaration of Helsinki. Written informed consent was provided by all participants of the study following institutional guidelines.

### Organoid generation and viability assays

Gefitinib-resistant NSCLC patient-derived organoids were generated by the fine mincing and enzymatic digestion of tumor tissues, followed by embedding in Matrigel (Corning, USA) and culturing in a specialized medium containing growth factors and inhibitors. The mammosphere formation capacity was evaluated by seeding dissociated organoid cells in plates with growth factors and assessing the formation of free-floating spherical clusters after 7 days. This integrated approach allowed for the comprehensive characterization of gefitinib-resistant NSCLC organoids and their response to various treatments.

### Quantitative qRT-PCR

The extraction of the total RNA from NSCLC cells was carried out while using TRIzol reagent (Invitrogen) and following the instructions given by the manufacturer. The synthesis of cDNA was carried out while employing reverse transcription from RNA (1 µg) using the PrimeScript RT Reagent Kit (Takara). Quantitative real-time PCR (qRT-PCR) was conducted employing SYBR-Green Master Mix (Takara) on a Bio-Rad CFX96 system, with gene expression normalized to a housekeeping gene. The 2^ΔΔCt^ method was used to analyze relative expression .The primer list is shown in Table S1.

### Plasmids and transfection

Lentiviral vector plasmids (shNAT10, shFATP4, shCPT1A, shP300, and NAT10-OE) were purchased from Tsingke (Beijing, China). The negative control included shNC plasmid or empty vector. As directed by the manufacturer, Lipofectamine 3000 (Invitrogen, USA) was used for transient transduction. Total RNA was collected for qRT-PCR analysis following a 48-hour culture. For lentiviral transduction, a second-generation lentiviral packaging system composed of psPAX2 and PMD2.G was used to create virus particles. The RNA oligonucleotide sequences. is shown in Table S2.

### Cell viability assays

NSCLC cell lines (H1975, H1650, PC-9, HCC2279, and PC-9/GR) were seeded in 96-well plates at 2000 cells/well density to evaluate cell viability. Each well contained 100 µL of complete medium. The cells were allowed to attach overnight. Varying concentrations of gefitinib or osimertinib, alone or in combination with the epigenetic inhibitor Remodelin, were then introduced. The vehicle DMSO (Solarbio, China) was administered to the control group.

Following the specified incubation duration, 10 µL of CCK-8 reagent (Dojindo, Japan) was introduced to each well, and the cells were incubated at 37 °C for 2 h. Absorbance was quantified at 450 nm with a microplate reader (BioTek, USA) to assess cell viability. The studies were independently conducted three times, with each treatment being carried out in triplicate. The mean ± standard deviation (SD) was used to express the results of calculating cell viability concerning the untreated controls.

### Cell cloning and mammosphere formation assays

NSCLC cells were seeded at a density of 1000 cells per well in 6-well plates for the colony formation test. The cells were treated to specific drug doses for 12 days following a 24-hour incubation period at 37 °C. After the procedure, cells were subjected to fixation with paraformaldehyde (4%, Solarbio, China) for 15 min, followed by their staining with crystal violet (0.1%, Beyotime, China), and rinsed with PBS. Excess stain was removed, and colony images were captured using a camera. Only colonies containing 50 or more cells were counted.

NSCLC cells were cultured at a density of 1 × 10^5^ cells/mL in serum-free DMEM/F12 medium (Gibco) with added growth factors such as EGF, bFGF, insulin, and BSA in ultra-low-attachment 6-well plates (Corning, USA) for the mammosphere formation experiment. A light microscope was used to view and count the mammospheres after the cells were cultured for 14 days to allow for their production.

### Cell apoptosis assay

Following the manufacturer’s instructions treated NSCLC cells were examined using the Annexin V-FITC/PI Apoptosis Detection Kit (BestBio, China) to measure cell apoptosis. In summary, cells were extracted using trypsinization without EDTA followed by centrifugation for 3 min at 1000 g. Following resuspension of the cell pellet in PBS, 1 × 10⁵ cells were added to 400 µL of binding buffer. After adding 5 µL of Annexin V-FITC and 10 µL of propidium iodide (PI), the mixture was allowed to sit at room temperature for 10 min in the dark. Flow cytometry (CytoFLEX, Beckman Coulter, USA) was used to evaluate the labeled cells, and FlowJo_V10 software was used to interpret the data on apoptotic cells.

### Dual-luciferase reporter assay

Through substituting T for C at the anticipated ac4C modification sites, site-directed mutagenesis was carried out to examine the function of ac4C modification on mRNA stability. Tsingke Biotechnology (Guangdong, China) produced both wild-type and mutant plasmids. For the luciferase reporter assay, co-transfection of PC-9/GR cells was carried out with the wild-type or mutant reporter constructs and a Renilla luciferase plasmid using Lipofectamine 2000 (Invitrogen). Following the manufacturer’s instructions, luciferase activity was assessed using a Dual-Luciferase Reporter Assay Kit (Beyotime) 48 h after transfection. To calculate the transfection efficiency, firefly luciferase activity was normalized to Renilla luciferase activity. Relative luciferase activity was used to record the outcomes of each experiment, which was conducted in triplicate.

### RNA sequencing

As directed by the manufacturer, TRIzol reagent (Invitrogen) was employed to separate total RNA from NSCLC cells. In order to ensure the quality of the samples, a NanoDrop spectrophotometer (Thermo Fisher, USA) was used to measure the RNA concentration and integrity after extraction. The NEBNext Ultra II RNA Library Prep Kit (New England Biolabs, USA) was then used to create RNA sequencing libraries. An Illumina HiSeq 3000 platform (Illumina, USA) was used to sequence the constructed libraries, producing paired-end 150 bp reads. The HISAT2 aligner was used to map the resultant sequences to the human genome reference (GRCh38/hg19), and DESeq2 was used to examine variations in gene expression.

### RNA stability assay

NSCLC cells were treated with actinomycin D (5 µg/mL) to inhibit transcription in the RNA stability test preparation. TRIzol reagent (Invitrogen) was used to extract RNA at 0, 4, 8, 12, and 24 h. A PrimeScript RT reagent kit (Takara, Japan) was employed for reverse transcription of the RNA to cDNA. SYBR-Green Master Mix (Takara) was utilized for qRT-PCR to detect mRNA levels, and the 2^ΔΔCt^ technique was used to evaluate RNA stability.

### Lipid droplet staining

NSCLC cells underwent the prescribed treatment after being plated in 6-well plates. After 15 min of being fixed with 4% paraformaldehyde, the cells were subjected to washing with PBS. Cells were treated with BODIPY 493/503 (1 µg/mL) for 30 min in the dark before being rewashed with PBS for lipid droplet staining. DAPI (1 µg/mL) was used to label the nuclei for 5 min. A microscope was used to take fluorescent pictures, and ImageJ was used to measure the amount of lipid droplets based on the BODIPY fluorescence signal.

### Western blotting

RIPA buffer with phosphatase and protease inhibitors (Beyotime, China; Roche, Switzerland; Bimake, USA) was used to lyse cells on ice. After combining protein samples with two SDS loading buffers and boiling them, the SDS-PAGE was separated, and the samples were transferred to nitrocellulose membranes (Cytiva, USA). NAT10 (Proteintech, China), FATP4 (Proteintech, China), CPT1A (Proteintech, China), SLC7A11 (Proteintech, China), and β-actin (Abcam, UK) primary antibodies were used to incubate the membranes after they had been blocked with 5% non-fat milk. The protein signals were then detected using ECL reagents (Advansta, USA), and imaging was carried out using a Bio-Rad ChemiDoc MP system after incubation with secondary antibodies (Proteintech, China). More antibody information is shown in Table S3.

### Immunohistochemistry (IHC)

Glass slides were plated with 4 μm slices of formalin-fixed, paraffin-embedded (FFPE) NSCLC tissue. The retrieval of Heat-induced antigen was performed in citrate buffer (pH 6.0) after deparaffinization and rehydration. 3% hydrogen peroxide inhibited endogenous peroxidase activity, and 5% BSA was used to reduce non-specific protein binding.

Primary antibodies against NAT10, FATP4, and CPT1A (Proteintech, China) were incubated in sections for an entire night at 4 °C. Following washing, the sections were left at room temperature for one hour to incubate with a secondary antibody (Abcam, UK). Visualization was performed using a DAB substrate kit (Dako, USA), followed by counterstaining with hematoxylin. Slides were then dehydrated, cleared, and coverslipped with a synthetic resin. Imaging and photography were carried out using the AMAFD1000 imaging system (Thermo Fisher).

### Seahorse assay

The Seahorse XF96 Analyzer (Agilent Technologies, USA) was used to measure the OCR and extracellular acidification rate (ECAR) to evaluate mitochondrial function. Seahorse XF96 plates were seeded with 1 × 10^5^ H1650 and H1975 NSCLC cells per well, and the cells were left to adhere for the entire night. The following day, the cells were washed and incubated in Seahorse XF Base Medium with 1.5 µM Oligomycin, 2 µM FCCP, and 0.5 µM ROT&AA for 1 h. According to the manufacturer’s instructions, the XF Cell Mito Stress Test Kit (Agilent Technologies) was used to evaluate mitochondrial parameters, including basal respiration, ATP production, and maximal respiration.

### LC-MS/MS analysis

Following collection, NSCLC cells were lysed in a 4:1 methanol: water ratio and rinsed with PBS. The supernatants were collected, dried, and reconstituted in 80% methanol following a 10-min, 12,000-g centrifugation at 4 °C. An Agilent 1290 LC system and a Q Exactive™ Plus mass spectrometer were used to filter and analyze the samples using LC-MS/MS. A mobile phase gradient of 0.1% formic acid in water and acetonitrile was employed for separation using a C18 column. Both positive and negative ion modes of mass spectrometry were used, and Xcalibur software was used to analyze the data and identify the metabolites.

### Acetylated RNA Immunoprecipitation sequencing (acRIP-seq)

Total RNA was isolated from NSCLC cells using TRIzol (Invitrogen) for acRIP-seq, and its quality was assessed using spectrophotometers the Agilent 2100 and NanoDrop 2000. By incubating 100 µg of RNA with fragmentation buffer at 70 °C for 5 min and then quickly cooling on ice, RNA samples with a RIN > 7.0 were broken up into 200–500 nucleotide segments. Anti-acetylcytidine (ac4C) antibodies attached to Dynabeads Protein G were then used to immunoprecipitate the fragmented RNA overnight at 4 °C. Following washing, Proteinase K was used to elute the RNA-protein complexes, and the RNeasy Mini Kit (Qiagen, Germany) was employed for the process of RNA purification.

The NEBNext Ultra II RNA Library Prep Kit was applied to prepare the library from immunoprecipitated and input RNA, and the Illumina NovaSeq platform was employed for sequencing. HISAT2 was used to analyze and align the reads to the human genome (hg38), and MACS2 was used to identify the ac4C peaks. The edgeR package in R was used to perform differential ac4C alteration analysis, while KEGG and GO analyses were used to evaluate pathway enrichment.

### Chromatin Immunoprecipitation (ChIP)

NSCLC cells (H1650 and H1975) were cultured to 80% confluence for the chromatin immunoprecipitation (ChIP) test. They were then treated with 1% formaldehyde for cross-linking and quenched with glycine. Cells were lysed, and the chromatin was sonicated into fragments ranging from 200 to 500 bp. The fragmented chromatin was incubated overnight with antibodies against p300 (Abcam) or H3K27ac (CST), then captured using Protein A/G magnetic beads (Thermo Fisher). The bound protein-DNA complexes were eluted after extensive washing, and overnight heating at 65 °C reversed the cross-links. The QIAquick PCR Purification Kit (Qiagen) was used to purify the DNA, and NAT10 promoter region-specific primers were used for qPCR analysis.

### Animal

The Ningbo University Animal Ethics Committee approved all animal experimentation (Approval NO. 13697). The male BALB/c nude mice were purchased from Beijing Vital River Laboratory Animal Technology Co., Ltd at four weeks of age.

In the shNAT10 subcutaneous tumor model, stable H1975 cells (6 × 10^6) expressing either shCtrl or shNAT10 were injected into nude mice. When the tumor size reached approximately 75–100 mm³, the mice were administered either an oral control drug, 25 mg/kg of Gefitinib, or 25 mg/kg of Osimertinib daily.

In the rescue experiment utilizing the NAT10 subcutaneous tumor model, stable H1975 cells (6 × 10^6) expressing shCtrl, shNAT10, shNAT10 & OE-FATP4, and shNAT10 & OE-CPT1A were injected into nude mice. When the tumor size reached approximately 75–100 mm³, the mice were administered either an oral control drug or 25 mg/kg of Gefitinib.

The nude mice’s right flank was subcutaneously injected with cell groups (6.0 × 10^6^ cells/100µL) suspended in PBS. The mice were either oral vehicle control or gefitinib (25 mg/kg) or Remodelin (100 mg/kg) daily once the tumor volume had grown to 75–100 mm³. Every two days, the tumor’s width (W) and length (L) were measured using calipers to calculate its volume using the formula V = (L × W²)/2. Tumors were removed for further analysis after the mice were euthanized.

### Statistical analysis

Prism 8 software (GraphPad Software, USA) was used to analyze the data. As shown in the figure legends, statistical analysis was conducted using either the two-way analysis of variance (ANOVA) or the unpaired two-tailed Student’s t-test, followed by Tukey’s multiple comparison post-tests.

## Supplementary Information

Below is the link to the electronic supplementary material.

Supplementary Material 1

## Data Availability

All data needed to evaluate the conclusions in the paper are present in the paper or the Supporting Information. The data that support the findings of this study are available from the corresponding author upon reasonable request.
