# Supplementary material for: NAT10-mediated lipid metabolic reprogramming drives EGFR-TKI resistance in non-small cell lung cancer via ac4C-dependent mRNA stabilization
Source: Exp Hematol Oncol. 2025 Nov 27;14:134. doi: 10.1186/s40164-025-00721-9 (PMC12661735; doi:10.1186/s40164-025-00721-9)
Supplement: Supplementary file 1 — Supplementary Material 1 [file 40164_2025_721_MOESM1_ESM.docx]

Supporting Information

**NAT10-Mediated Lipid Metabolic Reprogramming Drives EGFR-TKI Resistance in NSCLC via ac4C-Dependent mRNA Stabilization**

Shuai Fang^1†^, Yuchao Zhu^2†^, Wei Chen^1,3†^, Wei Mao^7†^, Yuan Fang^5^, Ziyuan Chen^1,3^, Zhiqi Hong^1,3^, Xiaodong Zhao^1^, Wenmin Su^1^, Yuning Pan^2^, Guangyu Yao^6✉^, Jianhua Wang^4✉^, Chengwei Zhou^1,3✉^

**Supplementary figures**

**
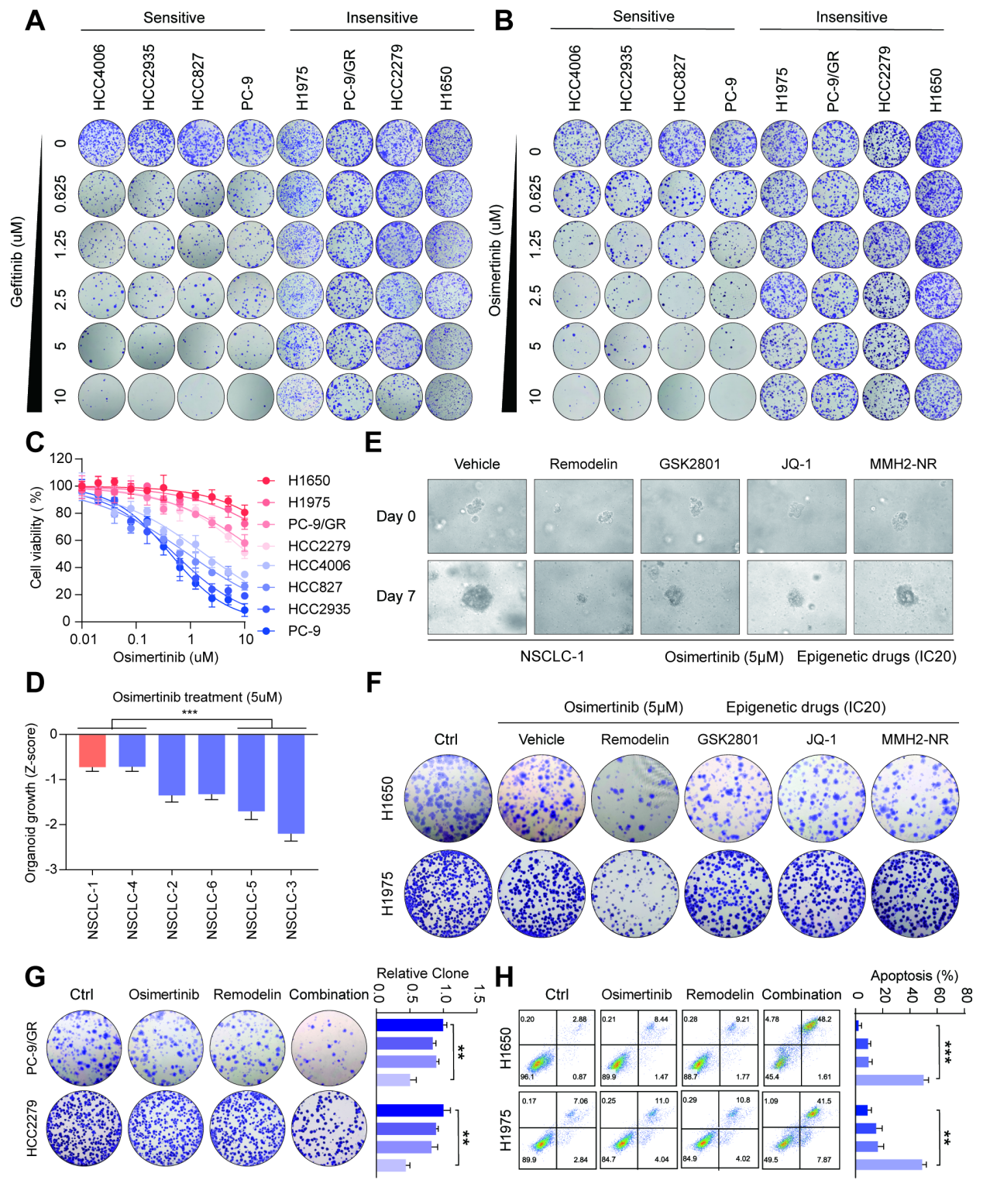
**

**Figure S1. Remodelin as a sensitizer for NSCLC-TKI therapy**

(A-B) Clonogenic assay comparing the effects of gefitinib and osimertinib on the colony formation ability of various NSCLC cell lines. (C) CCK-8 assays evaluating the impact of increasing concentrations of osimertinib on the viability of various NSCLC cell lines. (D) The effect of 5 μM osimertinib on the growth of NSCLC organoids after 7 days of treatment. (E-F) Evaluation of the combined effect of 5 μM osimertinib and IC20 concentrations of various epigenetic inhibitors on the growth of NSCLC-1-1 organoids and NSCLC cell lines. (G-H) Clonogenic assay results and flow cytometry analysis showing the effects of osimertinib, Remodelin, and their combination on the proliferation and apoptosis of resistant cells. Means ± SD of n = 3 independent experiments, two-tailed unpaired Student’s t-test. ****P* < 0.001; ***P* < 0.01; **P* < 0.05.

**Figure S2**

**
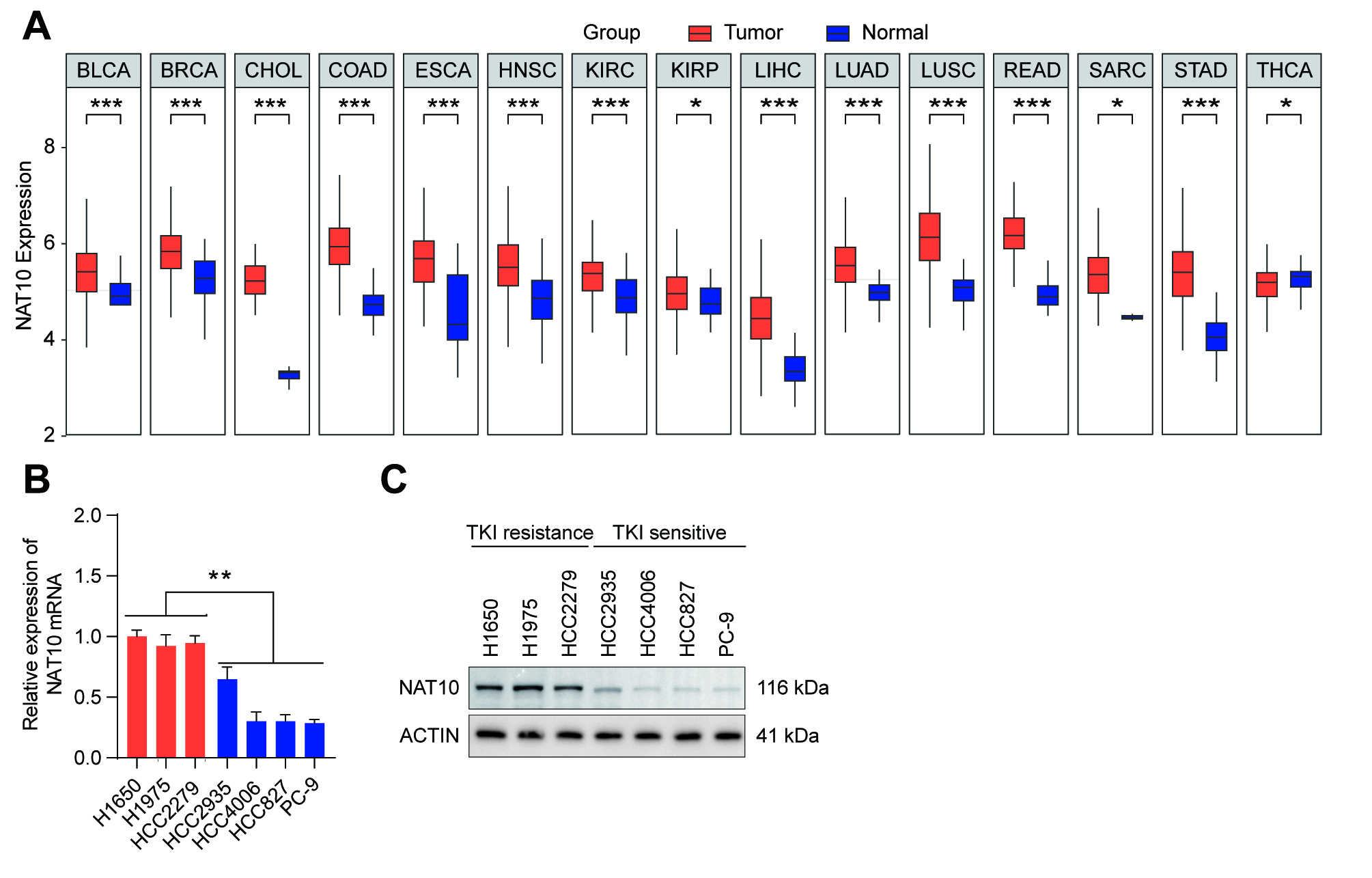
**

**Figure S2. Expression levels of NAT10 in NSCLC and its clinical significance**

(A) Analysis of NAT10 mRNA expression levels across various tumor types based on TCGA data. (B) Assessment of NAT10 expression in a panel of NSCLC cell lines using qRT-PCR analyses. (C) Western blotting analysis of NAT10 protein expression in different cell types. Means ± SD of n = 3 independent experiments, ****p < 0.0001, two-tailed unpaired Student’s t-test. ****P* < 0.001; ***P* < 0.01; **P* < 0.05.*

**Figure S3**


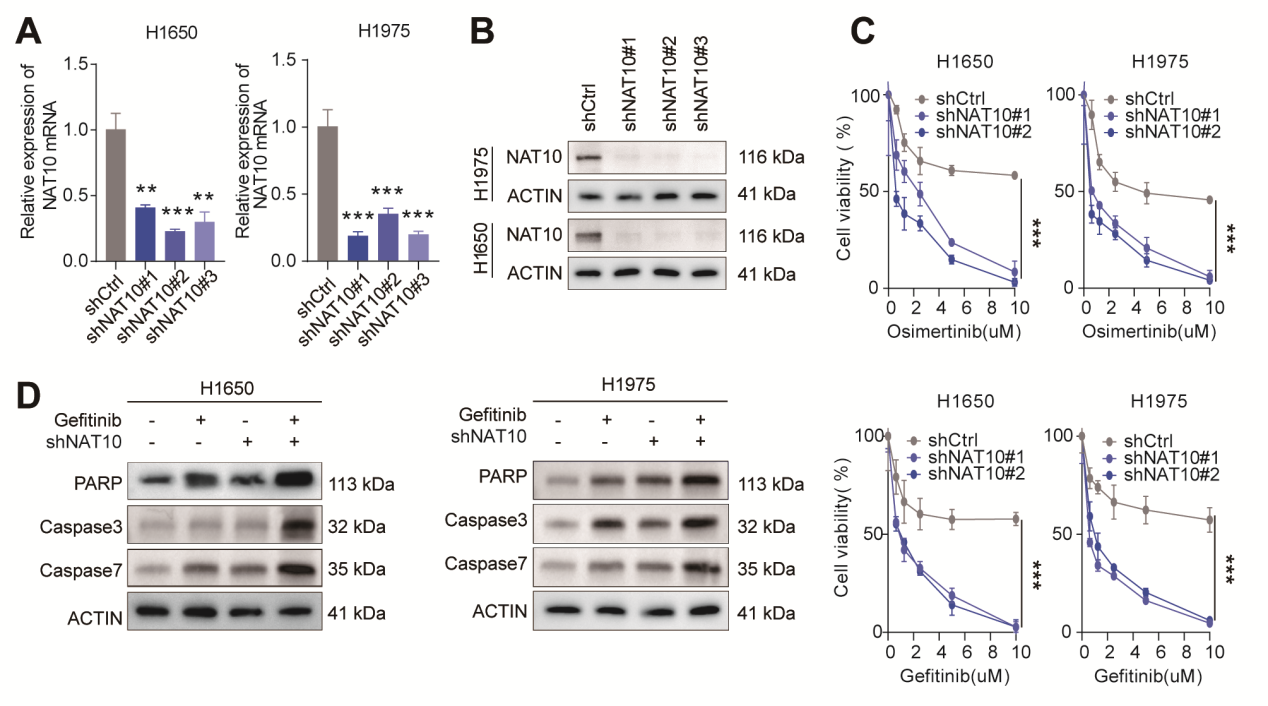


**Figure S3. Knockdown of NAT10 inhibits cell proliferation and enhances sensitivity to TKI therapy**

(A-B) qRT-PCR and Western blotting analyses of NAT10 expression in H1650 and H1975 cells transfected with shRNA targeting NAT10 (shNAT10) or the non-targeting control. (C) CCK-8 assays measured cell viability in H1650 and H1975 cells transfected with shNAT10 or shCtrl and treated with gefitinib or osimertinib. (D) Assessment of the expression of apoptosis-related proteins, as shown by Western blotting. Means ± SD of n = 3 independent experiments, ****p < 0.0001, two-tailed unpaired Student’s t-test. ****P* < 0.001; ***P* < 0.01; **P* < 0.05.

**Figure S4**


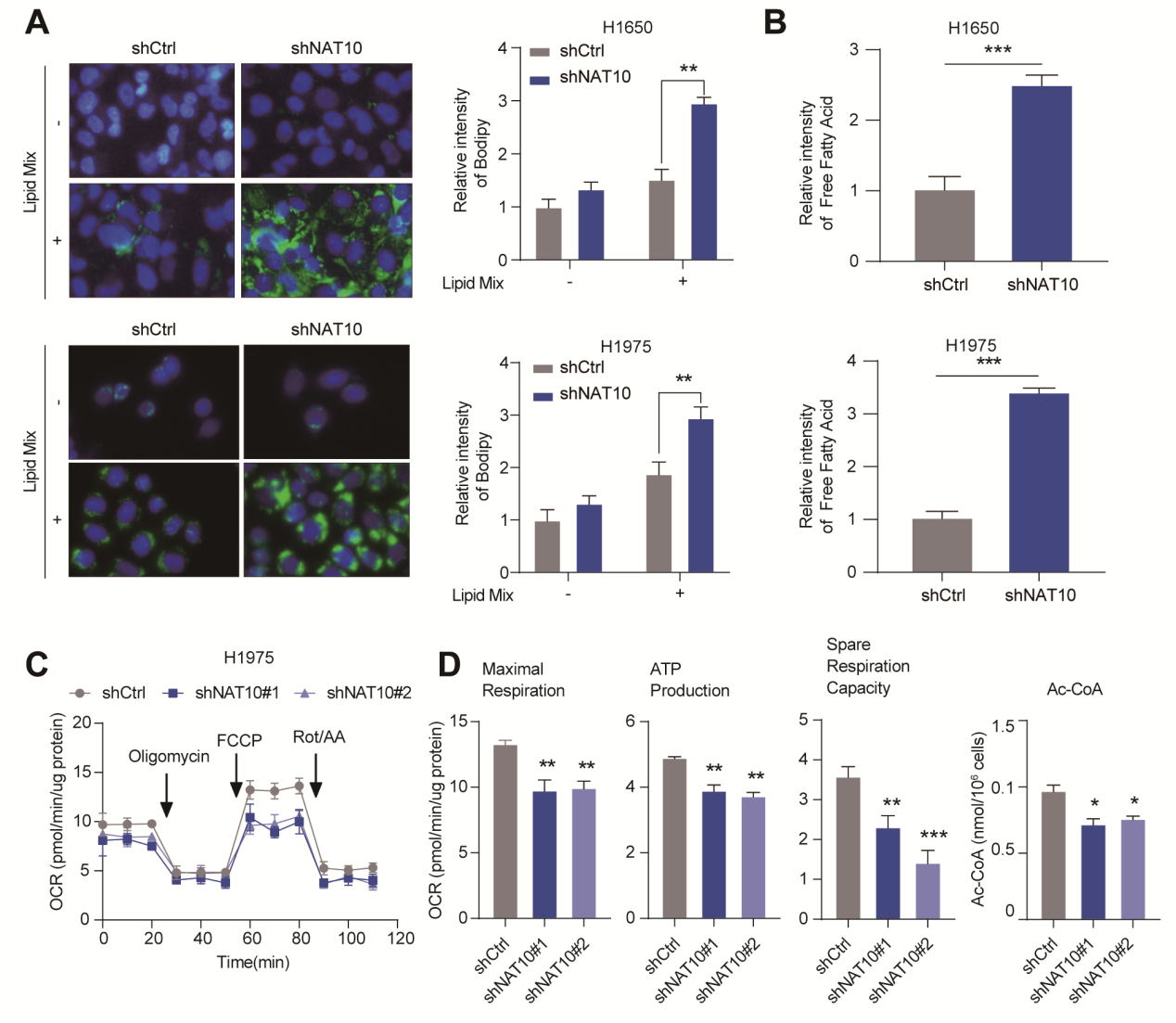


**Figure S4. NAT10 regulates lipid metabolism reprogramming in NSCLC**

(A) Lipid droplet staining was performed to evaluate lipid uptake after NAT10 knockdown in H1650 and H1975 cells. (B) Measurement of free fatty acid levels in NAT10-knockdown cells. (C-D) Effect of NAT10 knockdown on the mitochondrial respiratory parameters OCR, basal respiration, maximal respiration, ATP production, and spare respiratory capacity in H1975 cells, as measured by a cell flux analyzer. Means ± SD of n = 3 independent experiments, ****p < 0.0001, two-tailed unpaired Student’s t-test. ****P* < 0.001; ***P* < 0.01; **P* < 0.05.

**Figure S5**


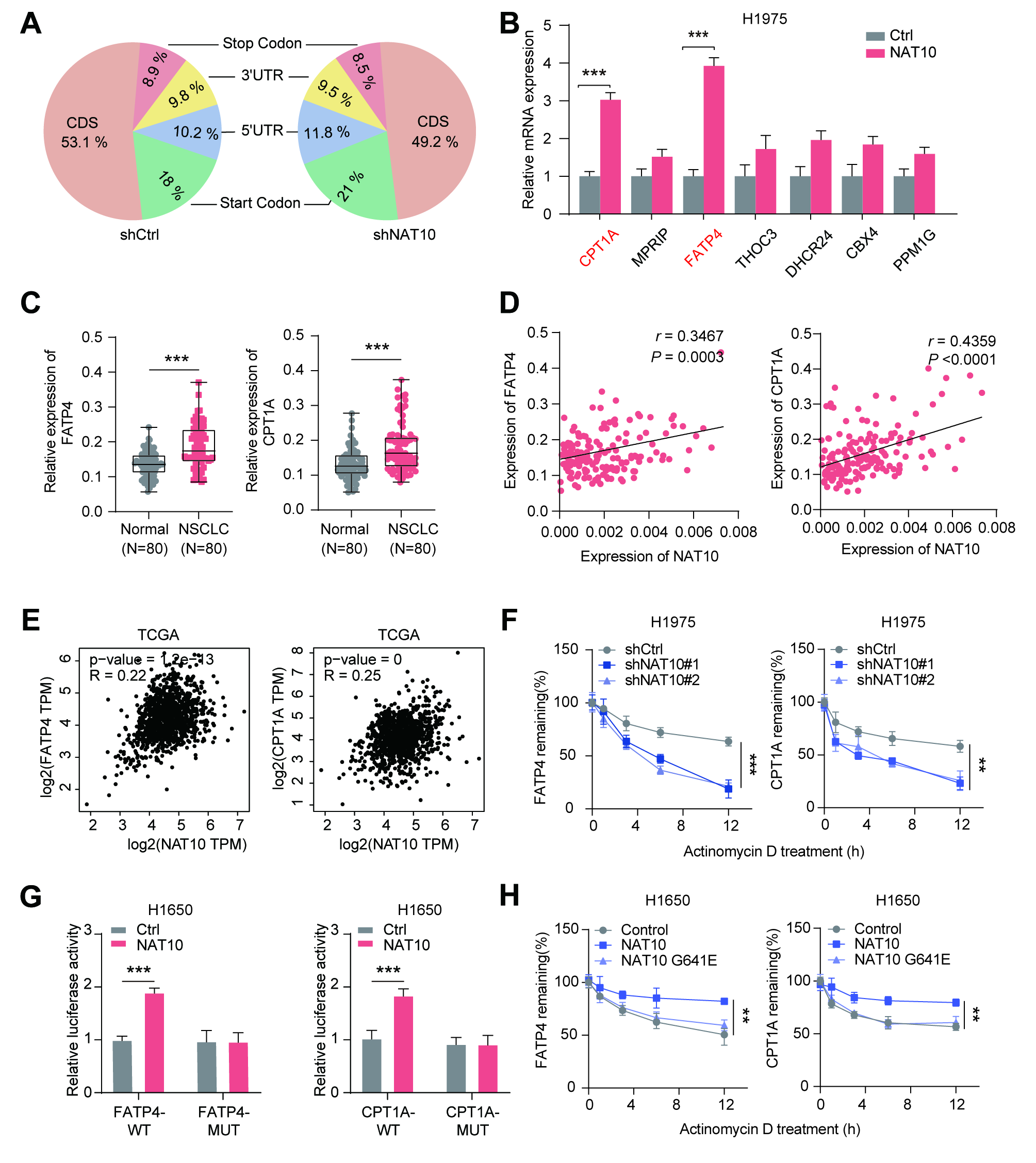


**Figure S5. NAT10 regulates the stability of FATP4 and CPT1A mRNAs via ac4C modification.**

(A) Genomic distribution of ac4C peaks in RNA transcripts. (B) Expression of common differentially expressed genes after NAT10 overexpression. (C) FATP4 and CPT1A expression in paired NSCLC and normal tissues. (D) Correlation of NAT10 expression with FATP4 and CPT1A expression in clinical samples. (E). Analysis of the correlation between NAT10 expression and FATP4 and CPT1A expression in the TCGA database. (F) FATP4 and CPT1A expression levels at different time points following actinomycin D treatment. (G) Luciferase reporter assay results showing the activity of reporter genes containing NAT10-binding regions or mutant sequences after NAT10 overexpression. (H) qPCR analysis of FATP4 and CPT1A expression levels following NAT10 or NAT10 G641E overexpression after actinomycin D treatment. Means ± SD of n = 3 independent experiments, ****p < 0.0001, two-tailed unpaired Student’s t-test. ****P* < 0.001; ***P* < 0.01; **P* < 0.05.

**Figure S6**


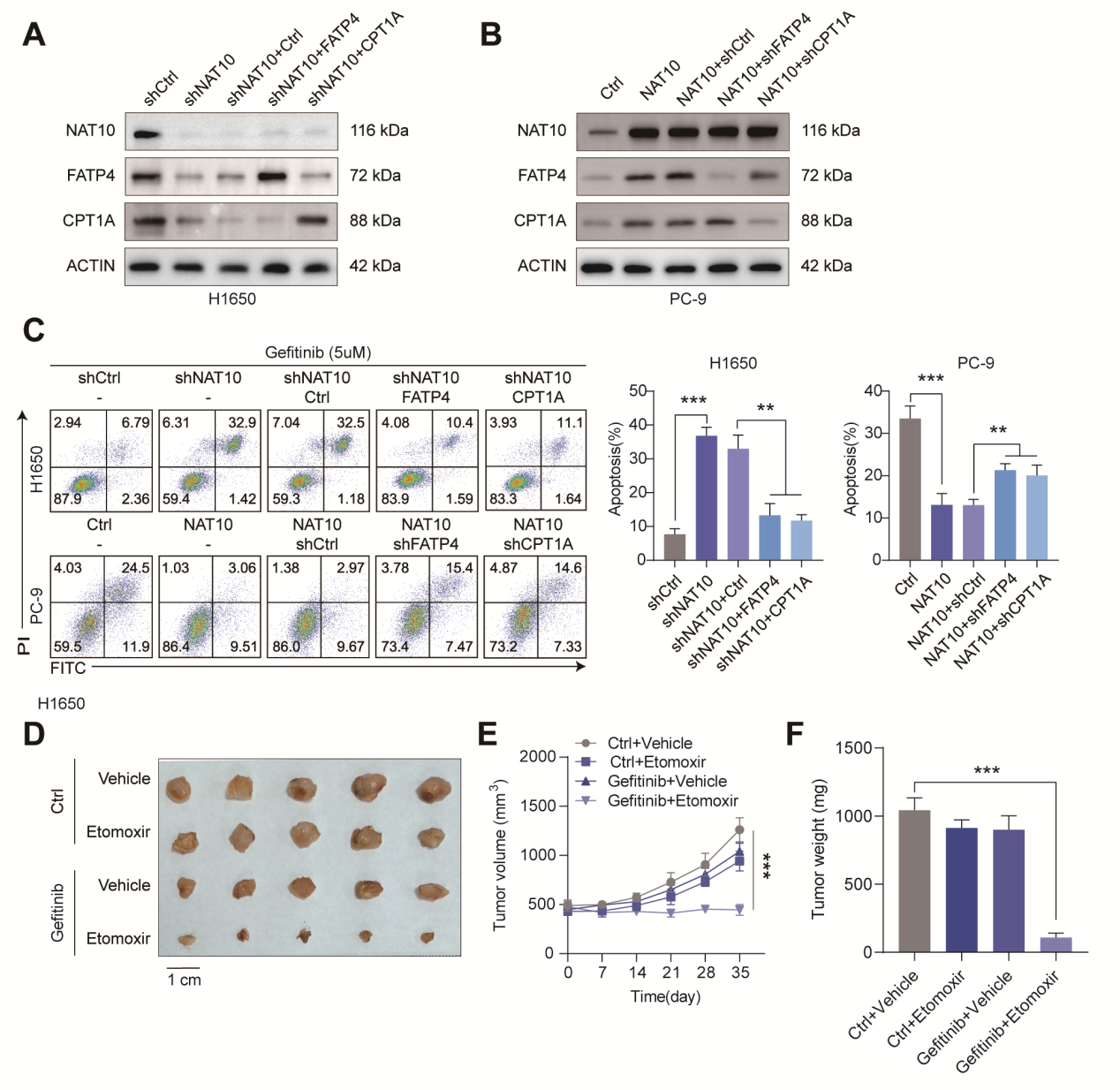


**Figure S6. NAT10 promotes TKI resistance in NSCLC cells by regulating FATP4 and CPT1A**

(A) Western blots showing NAT10, FATP4, and CPT1A protein expression in NAT10-knockdown cells with concurrent overexpression of FATP4 or CPT1A. (B) Western blotting shows the expression of NAT10, FATP4, and CPT1A proteins in NAT10-overexpressing cells with simultaneous knockdown of FATP4 or CPT1A. (C) Flow cytometry analysis of apoptosis in H1650 and PC-9 cells. (D) Subcutaneous xenograft model assessing the impact of the CPT1A inhibitor Etomoxir on tumor growth. (E-F) Tumor growth assessment in a subcutaneous xenograft model. Means ± SD of n = 3 independent experiments, ****p < 0.0001, two-tailed unpaired Student’s t-test. ****P* < 0.001; ***P* < 0.01; **P* < 0.05.

Figure S7


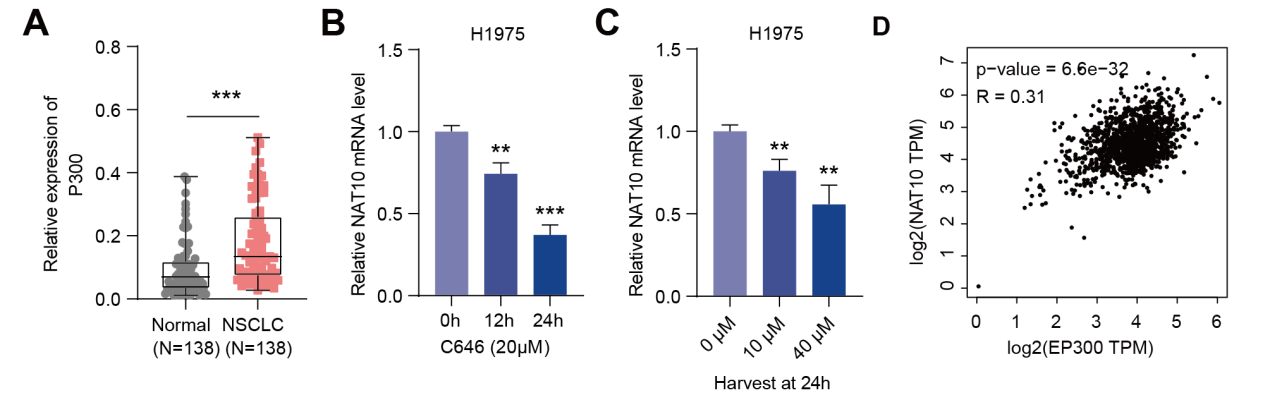


**Figure S7. p300-mediated H3K27ac activates NAT10 transcription in NSCLC**

(A) Verification of p300 expression in 138 tissue samples. (B) qRT-PCR analysis of NAT10 mRNA levels in H1975 cells treated with C646 (20 µM) at specified time points. (C) qRT-PCR analysis of NAT10 mRNA levels in H1975 cells treated with various concentrations of C646. (D) Correlations between NAT10 and p300 expression in NSCLC tissues. Means ± SD of n = 3 independent experiments, ****p < 0.0001, two-tailed unpaired Student’s t-test. ****P* < 0.001; ***P* < 0.01; **P* < 0.05.

**Figure S8**


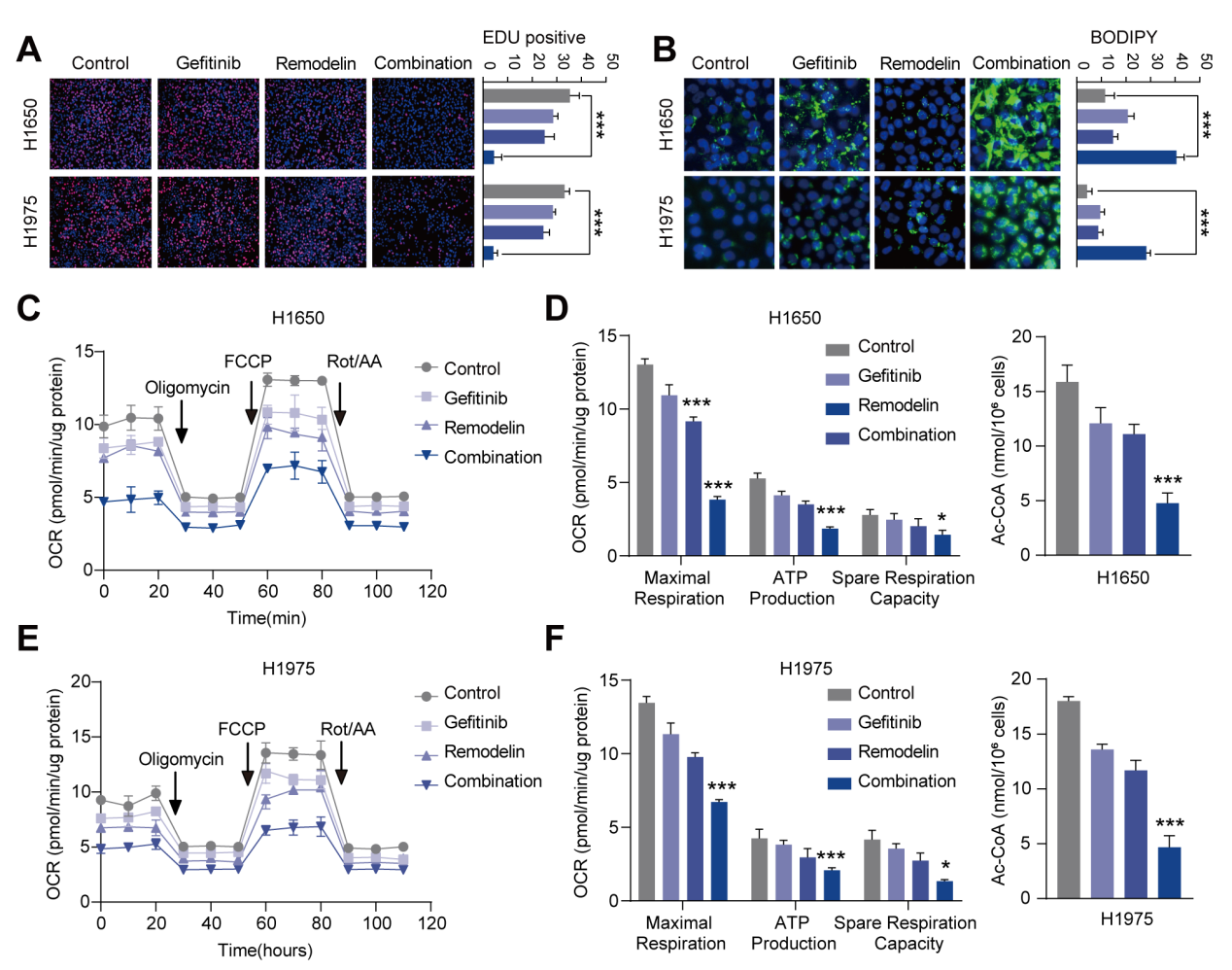


**Figure S8. Remodelin enhances NSCLC sensitivity to gefitinib in vitro and in vivo**

(A) Using EDU assays to determine cell proliferation after treatment with Remodelin, gefitinib, or their combination. (B) Lipid droplet staining for assessment of lipid metabolism levels. (C-D) Measurement of OCR in H1650 cells treated with Remodelin, gefitinib, or their combination to evaluate oxygen consumption rate, basal respiration, maximal respiration, ATP production, and spare respiratory capacity. (E-F) Measurement of the OCR in H1975 cells treated with Remodelin, gefitinib, or their combination to evaluate oxygen consumption rate, basal respiration, maximal respiration, ATP production, and spare respiratory capacity. * P < 0.05, ** P < 0.01, *** P < 0.001.

**Table S1 Primer sequences.**

| **Name** | **Forward primer (5’ to 3’)** | **Reverse primer (5’ to 3’)** |
| --- | --- | --- |
| NAT10 | GCCTCTTGTAAGAAGTGTCTCG | TCTTTTCAGAGATGCCCTCGAT |
| CD36 | GGGAAAGTCACTGCGACATG | TGCAATACCTGGCTTTTCTCA |
| FABP1 | GGAGGAATGTGAGCTGGAGACA | TATGTCGCCGTTGAGTTCGGTC |
| FATP4 | TGGCGCTTCATCCGGGTCTT | CGAACGGTAGAGGCAAACAA |
| FABP5 | TGAAGGAGCTAGGAGTGGGAA | TGCACCATCTGTAAAGTTGCAG |
| ATGL | CCATCACAGTGTCCCCCTTC | AACTGGATGCTGGTGTTGGT |
| MGL | AGGTGCCTACCATGTTCTCC | GTGGCTGTCCTTTGAGAGACC |
| HSL | AGGAGTCTGGGCCATAGCTTA | AGCACGCTAAAGCAGCAAAC |
| ACSL1 | GGGGGTATTCTTGCCTTCGT | TCAGCTGTGCGTGTTAGAGG |
| CPT1A | ATCAATCGGACTCTGGAAACGG | TCAGGGAGTAGCGCATGGT |
| CPT2 | CTGTAGCACTGCCGCATTCA | AGAGCAAACAAGTGTCGGTCAA |
| ACADL | TTGGCAAAACAGTTGCTCAC | ACATGTATCCCCAACCTCCA |
| ECHS1 | CTGTTACTCCAGCAAGTTCT | TCACACATCATGGCAAGCTCA |
| HADHA | AGTAGAAGCGGTGATTCCAGA | CCACGGGAGAGAAGTAGTGC |
| ACAA2 | CCATGGCAATGACTGCAGAG | GTAGCCAGCATCATTAGCAGC |
| MPRIP | AAAGCAACCCTGACTTCTTGA | CTCAACTTGGATGGGACACA |
| THOC3 | GTTGGCATCCAAGTAATCCTGA | TCTCCCCTTTAGTGTTCACAGT |
| DHCR24 | CCCACCCCAGCTGTAGTTAA | CAGCTTCCCTTACATGACGC |
| CBX4 | GCTGCTGATCGCCTTCCAGAAC | TTGGAACGACGGGCAAAGGTAG |
| PPM1G | CTGCTTCAGACTACCAAACTGG | CTCTGTAGAAATGGCAAACCACA |
| GAPDH | CGGAGTCAACGGATTTGGTCGTAT | AGCCTTCTCCATGGTGGTGAAGAC |

**Table S2 RNA oligonucleotide sequences.**

| **RNA oligos** | **Sequences** |
| --- | --- |
| sh-NC | Sense: 5’- CAACAAGATGAAGAGCACCAA -3’ |
| shNAT10#1 | Sense: 5’-CCTGTAATCTGTAGTTTAACA-3’ |
| shNAT10#2 | Sense: 5’-GAAATGTTATCATACTTTATT-3’ |
| shNAT10#3 | Sense: 5’AATTCAGGATTTAGAGACTGG--3’ |
| shFATP4#1 | Sense: 5’-AGGAGATAGAAGTGTTGTAGG-3’ |
| shFATP4#2 | Sense: 5’-AGTGTGATTTGTTGAACAAGC-3’ |
| shCPT1A#1 | Sense: 5’-GCATGTTTATTTCATTCTAAG-3’ |
| shCPT1A#2 | Sense: 5’-AGTCCTTTATGTTAAGAAAGG-3’ |
| shP300#1 | Sense: 5’-GGTTGATTCAAATAACTTAGG-3’ |
| shP300#2 | Sense: 5’-GCTGTTTAATACTCAGATATT-3’ |

Table S3 antibody information

| Name | Cat NO. |  |
| --- | --- | --- |
| CPT1A Polyclonal antibody | 15184-1-AP | Proteintech |
| SLC27A4/FATP4 Polyclonal antibody | 11013-1-AP | Proteintech |
| NAT10 Polyclonal antibody | 13365-1-AP | Proteintech |
| Histone H3 Polyclonal antibody | 17168-1-AP | Proteintech |
| Histone H3K27ac antibody | 39685 | Proteintech |
| p300 Polyclonal antibody | 20695-1-AP | Proteintech |
| PARP1 Polyclonal antibody | 13371-1-AP | Proteintech |
| Caspase 3/P17/P19 Polyclonal antibody | 19677-1-AP | Proteintech |
| Caspase 7 Polyclonal antibody | 27155-1-AP | Proteintech |
| Beta Actin Polyclonal antibody | 20536-1-AP | Proteintech |
